# VANET-GPSR+: A Lightweight Direction-Aware Routing Protocol for Vehicular Ad Hoc Networks

**DOI:** 10.3390/s26082525

**Published:** 2026-04-19

**Authors:** Zhuhua Zhang, Ning Ye

**Affiliations:** 1 School of Information and Control, Shenyang Institute of Technology, Shenyang 113122, China; 2 School of Computer Science and Engineering, Northeastern University, Shenyang 110819, China; yening1@cse.neu.edu.cn

**Keywords:** vehicular ad hoc networks (VANETs), Greedy Perimeter Stateless Routing (GPSR), direction awareness, link stability, probability model, routing optimization

## Abstract

Vehicular Ad hoc Networks (VANETs) feature high node mobility and volatile topologies, rendering the conventional Greedy Perimeter Stateless Routing (GPSR) protocol prone to weak link stability and inefficient route discovery due to its lack of direction awareness. Existing direction-aware improvements typically rely on multi-criteria weighting or clustering, introducing heavy parameter fusion and computational overhead that conflict with the resource-constrained nature of onboard units. To overcome these limitations, this paper presents VANET-GPSR+, a lightweight enhanced routing protocol. Its key novelty is that it discards multi-parameter fusion and relies solely on movement direction, supported by a synergistic framework of three lightweight mechanisms: direction-aware neighbor classification to prioritize nodes with consistent trajectories, adaptive greedy forwarding region expansion in sparse and dynamic networks, and path deviation angle-based next-hop selection. This work builds a probabilistic link lifetime model that theoretically quantifies the stability gains of direction awareness—a novel theoretical foundation. Comprehensive urban and highway simulations show that VANET-GPSR+ improves the packet delivery ratio by 16.3% and reduces end-to-end delay by 27.5% compared with standard GPSR, and it outperforms both OP-GPSR and AK-GPSR. It introduces negligible CPU and memory overhead, with CPU usage over 50% lower than the two benchmark protocols at 80 vehicles/km, and demonstrates strong robustness against varying beacon intervals and communication radii. Retaining GPSR’s stateless and distributed traits, VANET-GPSR+ delivers substantial performance gains with minimal overhead, serving as an efficient routing solution for highly dynamic VANETs.

## 1. Introduction

Intelligent Transportation Systems (ITS) have advanced rapidly in recent years, creating a strong demand for low-latency, high-reliability routing protocols in VANETs to support safety-critical applications including collision warning and cooperative driving [1,2]. As a foundational enabling technology for ITS, VANETs support efficient Vehicle-to-Vehicle (V2V) and Vehicle-to-Infrastructure (V2I) communications [3,4]. However, the inherent characteristics of VANETs—high node mobility, frequently fluctuating network topologies, and spatially heterogeneous traffic densities—present severe challenges to routing protocol design [5,6]. Furthermore, onboard units (OBUs) are subject to strict constraints in terms of hardware size, computation capability, and power consumption, resulting in limited resource budgets for protocol execution. Therefore, routing protocols must not only adapt to highly dynamic topologies but also meet strict lightweight constraints for onboard implementation.

Many routing protocols in VANETs are designed to address these challenges by leveraging network topology, vehicle position, service area, geographical location, and broadcasting mechanisms. Through these protocols, vehicles can provide drivers and passengers with real-time information, such as flooding updates, accident alerts, weather conditions, and traffic congestion reports. Equipped with this information, drivers and passengers can make informed decisions to avoid unwanted situations, thereby enhancing driving safety and overall travel experience [7].

Among various routing strategies, position-based protocols [8,9] have attracted extensive attention owing to their strong adaptability to dynamic topologies. By employing geographic position information for next-hop forwarding decisions, these protocols eliminate the heavy overhead of periodic route discovery and maintenance required in conventional topology-based routing, making them more compatible with the dynamic nature and resource constraints of vehicular networks. Nevertheless, existing position-based routing solutions still exhibit notable performance bottlenecks and fail to simultaneously achieve robust stability in highly dynamic scenarios and efficient lightweight operation for resource-limited onboard devices.

Standard GPSR and its later variants—including both multi-criteria decision-making and clustering-based designs—have focused largely on boosting packet delivery ratio and reducing end-to-end delay. Yet they have systematically neglected the co-design requirement of balancing routing performance with resource efficiency. As a result, no existing GPSR-derived approach can reconcile the fundamental tension between the severe link instability caused by high-speed node mobility and the stringent lightweight constraints of onboard units (OBUs). This gap motivates the need for a new direction-aware routing protocol that achieves high performance without incurring excessive overhead. These limitations are detailed in Section 2.

To address the aforementioned challenges, this paper proposes VANET-GPSR+, a lightweight enhanced routing protocol. Its core design philosophy is to fully exploit the regularity of vehicle movement direction, thereby achieving targeted optimizations that address stability flaws in dynamic scenarios. The protocol remains lightweight and well-suited for resource-constrained onboard units (OBUs). The main contributions of this work are summarized as follows:

**Direction-Aware Neighbor Screening**: A lightweight mechanism is designed to dynamically identify neighboring vehicles and prioritize nodes with consistent motion trajectories, thereby establishing robust transmission links and mitigating interruptions caused by directional mismatches.

**Adaptive Greedy Forwarding Range**: The candidate forwarding range is appropriately expanded to enhance routing reachability in sparse and high-dynamic scenarios, where standard GPSR often fails.

**Path Deviation Angle-Based Next-Hop Selection**: This metric replaces the single shortest-distance rule in standard GPSR as the core selection criterion, generating more efficient and stable multi-hop paths.

**Probabilistic Link Lifetime Modeling**: A theoretical model is constructed to quantify the stability improvements conferred by the direction-aware design, providing a solid theoretical foundation for the protocol.

**Comprehensive Performance Validation**: Extensive simulations under typical urban and highway scenarios verify that VANET-GPSR+ outperforms standard GPSR and state-of-the-art protocols in terms of packet delivery ratio (PDR), end-to-end (E2E) delay, and network robustness under variable conditions.

**Figure 1:**
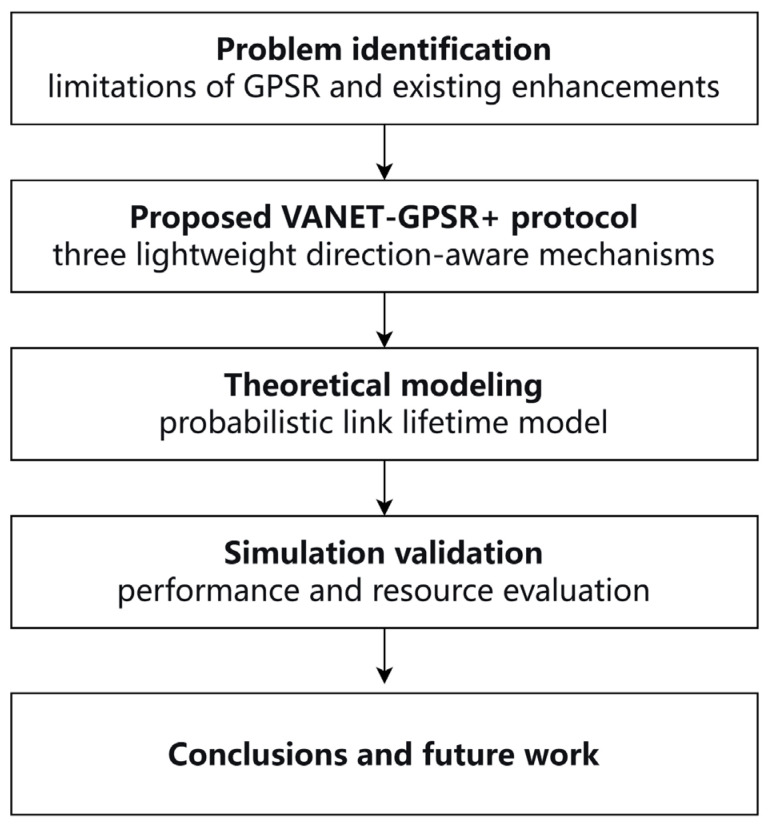
Research framework of VANET-GPSR+.

The overall research framework of this paper is illustrated in Figure 1.

The rest of this paper is arranged as follows: Section 2 introduces related works, Section 3 presents the detailed design of the VANET-GPSR+ protocol, Section 4 establishes a probabilistic link lifetime model, Section 5 describes the simulation setup and analyzes the experimental results, and Section 6 summarizes the work of this paper and outlines future research directions.

## 2. Related Work

### 2.1. Classic Protocol as Benchmark and Its Limitations

Position-based routing protocols have attracted considerable research attention owing to their inherent adaptability to dynamic network topologies. Among these, the GPSR protocol [10] serves as a critical benchmark, valued for its stateless architecture and minimal resource overhead. However, GPSR exhibits inherent limitations in VANET environments: its greedy forwarding strategy relies exclusively on instantaneous geographic distance, completely disregarding vehicle movement direction. This frequently results in abrupt link disruption when selected nodes move away rapidly. Furthermore, its rigid “distance-decreasing” rule often leads to routing failures in sparse network scenarios. These inherent flaws preclude GPSR from meeting the stability requirements of practical vehicular applications.

Subsequent studies have analyzed the impact of key parameters such as beacon interval and transmission rate on GPSR performance in urban scenarios [11], revealing that parameter optimization can significantly improve packet delivery ratio, particularly under high vehicle density conditions. However, such adjustments remain superficial fixes that fail to resolve the fundamental issue of direction neglect.

To overcome these limitations, recent research has focused on two dominant optimization paradigms: multi-criteria decision-making (MCDM) algorithms [12,13,14,15,16,17] that refine next-hop selection criteria, and clustering-based routing schemes [18,19,20,21] that restructure network topology. Although these methods improve performance in certain scenarios, they introduce unavoidable costs, such as higher computational complexity, excessive management overhead, and heavy reliance on real-time network state information. More importantly, they fail to fundamentally address GPSR’s core deficiency of neglecting vehicle movement direction, and their added complexity often conflicts with the resource-constrained nature of OBUs, which operate under strict limitations on computational power, memory, and energy consumption.

Beyond these two mainstream directions, some studies have proposed alternative enhancements that do not rely on multi-metric weighting or clustering. For instance, PA-GPSR [22] extends the neighbor table with two additional structures—a Deny Table (DT) and a Recently Sent Table (RST)—to avoid routing loops and reduce redundant forwarding. While this approach effectively eliminates routing loops and reduces path redundancy, it introduces non-negligible memory overhead due to the maintenance of the additional tables. Nevertheless, it demonstrates that GPSR optimization can take forms other than multi-metric weighting or clustering, thereby highlighting the diversity of potential improvement strategies for GPSR-based routing in VANETs.

In the following subsections, we review these two principal research threads in detail, analyzing their respective strengths and limitations with particular emphasis on their implications for lightweight protocol implementation in resource-constrained OBU environments.

### 2.2. Optimization Protocols with Multi-Criteria Decision-Making

MCDM protocols enhance GPSR by integrating diverse metrics—including link quality, node load, and mobility and so on—into the forwarding decision process.

OP-GPSR [12] serves as a representative MCDM-based enhancement, employing a multi-criteria cost function that accounts for geographic distance, neighbor movement state, current node load, and wireless link quality. Simulation results confirm that OP-GPSR outperforms standard GPSR in both PDR and end-to-end delay. However, its reliance on real-time multi-dimensional state information compels each node to periodically collect, process, and evaluate multiple parameters, imposing substantial computational burdens on resource-constrained OBUs.

MM-GPSR [13] improves GPSR’s greedy forwarding by introducing a cumulative communication duration metric and a defined allowed communication area, selecting the neighbor with the longest cumulative duration as the next hop. For perimeter forwarding, it adopts a minimal-angle criterion to mitigate path redundancy. While these modifications enhance routing stability, they necessitate parameter tuning (e.g., the *λ* factor governing the allowed communication area) and periodic cumulative duration calculations, increasing both computational load and memory overhead—critical constraints for resource-limited OBUs.

DVA-GPSR [14] selects relay nodes via a weighted function that integrates heading angle, speed variation, and node density, improving delivery rates but demanding periodic weight recalculation. Similarly, W-PAGPSR [15] integrates node distance, reliable node density, link duration, and movement direction into a weighted greedy forwarding strategy, boosting routing stability in sparse urban grids yet incurring additional computational overhead from continuous parameter collection and weight updates.

Recent extensions of MCDM approaches have sought to refine performance. WA-GPSR [16] computes a reliable communication area and selects next-hop vehicles based on multi-faceted routing criteria, outperforming both MM-GPSR and traditional GPSR. W-GPSR [17] introduces a Greedy Link Weight Factor (GLWF) that incorporates mobility prediction into forwarding decisions, effectively optimizing greedy forwarding in highly dynamic vehicular environments.

In summary, MCDM protocols enhance GPSR performance at the expense of elevated computational complexity. They remain dependent on multiple metrics and require periodic weight recalculation, which imposes significant overhead on resource-constrained OBUs. In contrast, our VANET-GPSR+ uses direction as the sole criterion and reuses existing beacons, incurring negligible overhead.

### 2.3. Topology Management Protocols Based on Clustering Learning

Clustering-based protocols reduce routing overhead by grouping vehicles with similar mobility patterns into clusters, where cluster heads (CHs) oversee intra- and inter-cluster communication. This clustering strategy mitigates the impact of dynamic topology changes in VANETs, complementing GPSR’s position-based forwarding to enhance routing reliability.

AK-GPSR [18] exemplifies this approach, employing an adaptive *K*-medoids unsupervised learning algorithm with the Gap statistic to determine the optimal cluster number (*K* value) and partition vehicles into stable clusters. GPSR is then adopted for intra- and inter-cluster packet forwarding, constructing a relatively stable virtual topology: as long as cluster structures remain intact, intra-cluster vehicle position changes do not trigger drastic routing table fluctuations. Theoretically, this reduces route discovery frequency and enables more reliable data transmission. However, maintaining cluster stability requires extensive signaling for CH election and member tracking—an issue exacerbated in high-speed urban traffic. Unreasonable cluster partitioning further induces convoluted routing paths and increased end-to-end delay.

Alternative clustering paradigms have been developed to further optimize GPSR performance. A hybrid GOA-WOA bio-inspired clustering algorithm [19] integrates Grasshopper Optimization and Whale Optimization, synthesizing speed, direction, position, and residual energy to generate stable cluster structures, with GPSR used to evaluate routing performance on these clusters. A mobility clustering-based roadside unit (RSU) deployment method [20] formulates RSU placement as a clustering problem, leveraging clustering results to optimize vehicle-to-RSU communication and validating deployment effectiveness via GPSR. Additionally, a hybrid scalability enhancement approach [21] combines mobility clustering with Bloom filters, prioritizing packet forwarding to CHs based on clustering outcomes before employing GPSR for efficient routing, which substantially reduces control overhead.

In summary, clustering protocols reduce routing overhead but introduce substantial signaling costs and exhibit sensitivity to topology changes. They fail to directly address GPSR’s core deficiency of neglecting vehicle movement direction, and their maintenance overhead often conflicts with the lightweight deployment requirements of resource-constrained OBUs.

Table 1 summarizes the key features and limitations of the representative GPSR-based protocols discussed above.

### 2.4. Positioning of This Work

Existing GPSR enhancements fall into two main categories: multi-parameter weighted fusion and network structure restructuring. Both improve certain performance metrics but introduce substantial computational or communication overhead, a critical limitation for resource-constrained OBUs.

VANET-GPSR+ adopts a fundamentally different design, with three key innovations:

**Metric simplification:** the design moves from multi-parameter to single-attribute design. Unlike fusing distance, speed, load, and link quality, VANET-GPSR+ depends exclusively on vehicle movement direction—the most critical and readily available attribute of VANET link stability. This eliminates weight tuning, multi-parameter storage, and complex calculations.

**Algorithmic synergy:** integrated closed-loop rather than incremental heuristics. The three proposed mechanisms form a direction-centered synergistic system, not isolated stacked rules: direction-aware neighbor classification filters stable candidates; adaptive greedy forwarding region expansion ensures sparse-network connectivity; path deviation angle selection optimizes path efficiency. Their joint operation achieves high performance with near-zero additional overhead, a feature absent from existing incremental GPSR variants.

**Theoretical grounding:** probabilistic validation of direction-aware stability. Unlike most heuristic-based improvements, this work establishes a probabilistic link lifetime model (Section 4) that rigorously quantifies how prioritizing consistent-direction vehicles extends link duration. This mathematical basis differentiates VANET-GPSR+ from empirical modifications.

VANET-GPSR+ embodies a new lightweight intelligence paradigm—achieving more with less. It retains GPSR’s stateless, distributed nature, extracts direction from existing periodic beacons, and incurs no extra control packets, complex computations, or memory-intensive state maintenance. This makes it inherently suitable for resource-constrained OBUs while delivering substantial performance gains (Section 5).

The next section details the design of VANET-GPSR+.

## 3. VANET-GPSR+ Routing Design

### 3.1. Direction-Aware Neighbor Classification

GPSR exhibits prominent performance limitations under high-vehicle-mobility scenarios. The protocol’s next-hop selection mechanism relies exclusively on the instantaneous geographic distance of neighboring nodes, without accounting for the movement direction of these nodes. In high-speed scenarios such as highways and dense urban road networks, the positional information used for routing decisions becomes obsolete in an extremely short time frame; a neighbor node that appears closest to the destination at the moment of decision-making may have moved significantly before packet transmission is completed.

**Figure 2:**
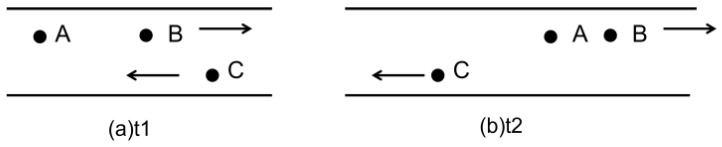
The shortcomings of traditional GPSR protocol. Node A, B, C are vehicles; arrows show movement directions.

As illustrated in Figure 2, this forwarding strategy inherently assumes a static network environment, which is inconsistent with the intrinsic dynamic characteristics of VANETs. Specifically, it presupposes that the network topology remains relatively stable within the neighbor information update cycle *T*. However, the high mobility of vehicle nodes results in a critical issue: the “optimal” next-hop node selected based on outdated positional information (e.g., at time *t*_1_, such as node C) may move out of the effective communication range by the actual packet transmission time (*t*_2_, where *t*_2_ − *t*_1_ < *T*) due to high-speed opposing or crossing movement. This phenomenon triggers instantaneous link disruption, packet loss, and frequent routing reconfiguration, which collectively degrade the overall performance of the protocol significantly.

To alleviate mobility-induced link instability, we propose a lightweight direction-aware neighbor classification mechanism. It identifies and exploits the regularity of vehicle movement direction to prioritize stable relays. A fundamental premise of this mechanism is that in road-constrained traffic environments, vehicles moving in approximately the same direction exhibit lower relative speeds and more stable topological relationships, thereby forming wireless links with longer expected lifetimes and higher reliability. Based on this premise, the proposed algorithm dynamically categorizes neighboring nodes into two priority sets: a high-priority same-direction neighbor list and a low-priority opposite-direction neighbor list.

The specific classification decision method is detailed as follows:

**Motion Vector Extraction.**

Each node periodically broadcasts beacon messages carrying its position information. Let the source node *S* record its own positions at two consecutive times as $\vec{S_{1}}$ and $\vec{S_{2}}$, and the positions of a specific neighbor node *N* as $\vec{N_{1}}$ and $\vec{N_{2}}$. The approximate motion vectors for *S* and *N* over time interval Δt can be expressed as:

(1) $$\vec{V_{S}}=\vec{S_{2}}-\vec{S_{1}},\;\vec{V_{N}}=\vec{N_{2}}-\vec{N_{1}}.$$

**Direction Angle Calculation.**

The direction consistency is quantified using angle *θ* between the two motion vectors. The angle between the source node vector and the neighbor node vector $\vec{V_{S}}$ and $\vec{V_{N}}$ is:

(2) $$\theta =\arccos\left(\frac{\vec{V_{S}}\cdot \vec{V_{N}}}{|\vec{V_{S}}|\cdot |\vec{V_{N}}|}\right).$$

where θ ranges from [0, π].

**Neighbor Classification.**

Based on the angle θ and a set judgment threshold, neighbor nodes are dynamically classified into different sets:

- If $\left.\theta \in [0,\pi /2\right)$, the neighbor node is considered a co-directional neighbor node of the source node. Such nodes have a smaller relative speed with *S*, indicating a high expected link stability.
- If θ∈[π/2,π], the neighbor node is considered a reverse-direction neighbor node of the source node. Such nodes have a larger relative speed with *S*, making the link prone to rapid interruption.

Many existing direction-aware routing protocols integrate multiple metrics—including speed, node density, and movement direction—into a weighted function. In contrast, the proposed classification mechanism relies solely on movement direction extracted from periodic beacon messages, which significantly reduces computational overhead. By prioritizing next-hop selection from the high-priority co-directional neighbor list, VANET-GPSR+ proactively avoids neighbor nodes that are geometrically close but moving away rapidly. This design enhances the persistence of single-hop links, reduces packet loss and retransmission delays, and minimizes control overhead caused by frequent topology changes. Notably, the mechanism only requires basic vector operations, which perfectly aligns with the requirements of lightweight, distributed algorithms in VANETs.

### 3.2. Adaptive Greedy Forwarding Region

Building on the direction-aware neighbor classification above, we further propose an adaptive greedy forwarding region expansion scheme to address routing failures in sparse or dynamic networks. In standard GPSR, a node can only forward packets to neighboring nodes that are closer to the destination than itself. This constraint restricts the next-hop search to a narrow geometric region. Under scenarios of sparse network density or rapid topology changes, this restricted region may occasionally contain no valid candidates. Consequently, GPSR is forced to switch to perimeter forwarding, a slower and more circuitous routing mode that often degrades overall protocol performance.

**Figure 3:**
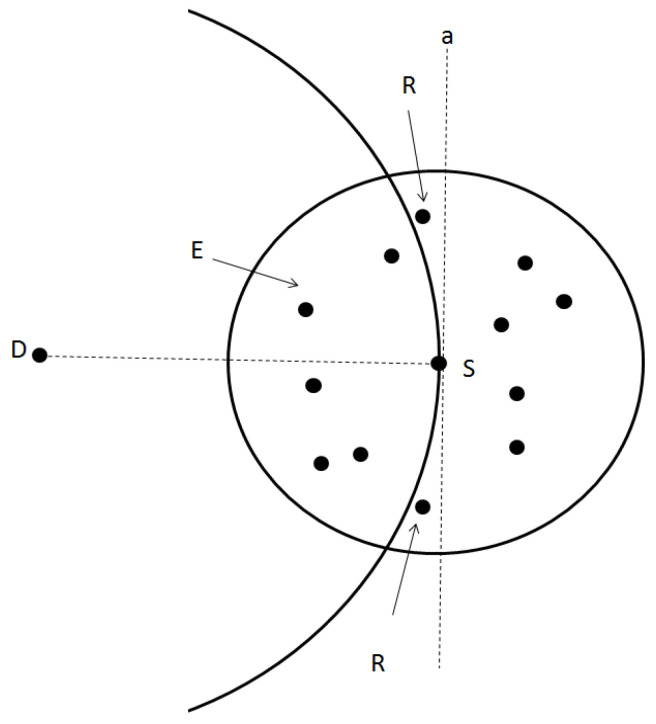
Candidate Node Selection Strategy for GPSR.

Figure 3 illustrates the geometric principle underlying this limitation. Suppose source node *S* intends to transmit a packet to destination *D*; the dark-colored nodes in the figure are assumed to move in the same direction as *S* for the sake of concreteness. GPSR’s core forwarding rule dictates that any relay node *N* must be closer to *D* than *S* is, which means *N* must lie within a circle centered at *D* with a radius equal to the distance between *S* and *D* (*SD*). Additionally, *N* must be within the radio communication range of *S*. The feasible candidate region for next-hop selection is the intersection of these two circles—the area *E* in Figure 3—which is relatively small in the illustrated scenario.

VANET-GPSR+ expands the candidate search scope. Instead of enforcing a strict requirement that each hop must move closer to *D*, it prioritizes neighboring nodes that are at least moving in the direction of *D*. The revised forwarding rule is defined as follows: a neighbor node *N* qualifies as a next-hop candidate if it resides in the half-plane containing *D*, on the opposite side of the perpendicular bisector of the line segment *SD*. In simpler terms, this region corresponds to the entire forward-facing portion of S’s communication circle, encompassing both region *E* and region *R* in Figure 3.

This expanded candidate set is denoted as $C_{greedy}$. Mathematically, *N* belongs to ${\;C}_{greedy}$ if $(\vec{N}-\vec{S})\cdot (\vec{D}-\vec{S})\geq 0$—the non-negative dot product indicates that *N* is not located behind *S* relative to the destination *D*.

This straightforward modification significantly increases the number of available next-hop candidates, which is particularly beneficial in sparse road traffic or high-mobility scenarios with random vehicle movements. Importantly, this enhancement requires no additional information: all necessary data is derived from the position information that nodes already exchange via regular beacon messages. As a result, the protocol retains its lightweight nature while achieving a much higher probability of finding a valid forward path.

### 3.3. Optimal Next-Hop Selection Based on Path Deviation Angle

Based on the expanded candidate set generated in the previous subsection, we define a new path deviation angle metric to select the optimal next-hop node with improved stability and path efficiency. Once the candidate set $C_{greedy}$ is determined, the protocol still needs to select a single node from this set for actual packet forwarding. In standard GPSR, the next-hop node is selected as the neighbor closest to the destination *D*, with the selection decision dominated solely by instantaneous geographic distance. However, in high-vehicle-mobility road scenarios, instantaneous distance is a poor indicator of link stability across subsequent forwarding hops. A neighbor node that is slightly farther from *D* but moving in a direction approximately consistent with the destination’s direction often proves to be a far more stable and reliable forwarding choice.

**Definition and Calculation of Path Deviation Angle.**

To address this limitation and select a more stable next-hop node from $C_{greedy}$, VANET-GPSR+ introduces the path deviation angle as its core next-hop selection criterion. Unlike the distance-dominated selection strategy in standard GPSR, this criterion focuses on evaluating the path smoothness introduced by selecting a candidate node, i.e., the degree of path bending if the packet is forwarded through that node. For any candidate node $N\in C_{greedy}$, two key vectors are defined: the forward vector $\vec{SN}=\vec{N}-\vec{S}$ and the destination vector $\vec{SD}=\vec{D}-\vec{S}$. The included angle between these two vectors, denoted as $\varphi_{NSD}$, quantitatively characterizes the degree of path bending. Mathematically, $\varphi_{NSD}$ is directly derived from the dot product operation, and its calculation formula is expressed as follows:

(3) $$\varphi_{NSD}=arccos\left(\frac{\vec{SN}\cdot \vec{SD}}{|\vec{SN}|\cdot |\vec{SD}|}\right).$$

**Geometric Meaning and Selection Strategy.**

The path deviation angle $\varphi_{NSD}$ quantifies the degree of detour of the forwarding path via candidate node *N* from the straight-line path *S→D*. A small $\varphi_{NSD}$ (approaching 0) indicates that *N* is approximately located on the direct line between *S* and *D*, ensuring that packets can maintain a forward direction and avoiding zigzag adjustments in subsequent forwarding hops to return to the optimal path. In contrast, a large $\varphi_{NSD}$ implies potential routing inefficiencies, such as path detours that may lead to routing dead ends or loops.

In practical implementation, the proposed algorithm first scans the high-priority co-directional neighbor list and selects the node with the smallest $\varphi_{NSD}$. If no valid candidates exist in this list, the algorithm then searches the remaining nodes in $C_{greedy}$. The core objective of this strategy is to maintain the most straightforward forwarding direction while prioritizing neighbor nodes with high link stability (i.e., nodes likely to remain within communication range), thereby guiding the overall routing path toward the Euclidean shortest path.

The effectiveness of this selection strategy primarily stems from its ability to reduce path tortuosity and improve end-to-end performance over multiple hops. The core benefit lies in maintaining a straighter forwarding direction, which shortens subsequent hop distances and keeps relative speeds low across the entire route. By selecting the co-directional neighbor node with the smallest $\varphi_{NSD}$ (i.e., the node pointing most directly toward the destination), the protocol achieves a forwarding path with minimal redundant motion. Recent research findings have confirmed that angle-aware routing strategies can effectively reduce path length and improve routing efficiency [15]. This advantage stands in stark contrast to the next-hop selection criterion of the original GPSR. Specifically, GPSR may select a neighbor node that is marginally closer to *D* but deviates significantly from the forward direction; such a node may lead the packet into a routing dead end or force a large U-turn in subsequent hops, thereby degrading routing performance. In contrast, the path deviation angle criterion effectively circumvents these drawbacks by inherently favoring nodes that maintain a smooth and straight forwarding path. Importantly, this enhancement requires no additional beacon exchange or signaling, as it uses only vector information from existing beacons, The only computational cost is a lightweight dot-product and an arccos operation per neighbor candidate, which adds only minimal computational overhead. As such, the proposed algorithm fully preserves its lightweight and computationally efficient nature.

## 4. Link Stability Modeling

To theoretically verify the rationality of the VANET-GPSR+ protocol design and provide solid mathematical support for the three core mechanisms proposed in Section 3, this section establishes a link lifetime probability model. This model is intended to characterize the probability that a wireless link between a pair of communicating vehicle nodes remains connected over time, with a focus on analyzing the decisive impact of relative movement direction and relative motion speed on the expected link lifetime. By correlating the derivation results of the model with the core design objectives of the VANET-GPSR+ protocol, a complete argumentation loop from theoretical modeling and mechanism design to subsequent experimental verification is formed, further consolidating the theoretical foundation of the protocol design.

### 4.1. Model Establishment

Consider source node *S* and one of its candidate next-hop nodes *N*. We make the following reasonable assumptions to establish an analyzable model, which are commonly adopted in vehicular link stability studies:

- Initial State: At decision time t = 0, both nodes are within each other’s communication range, with an initial Euclidean distance $d_{0}$, satisfying $0<d_{0}<R$, where *R* is the node’s communication radius [23].
- Relative Motion: Within a short time window (e.g., during one packet transmission), the motion of node *N* relative to node *S* can be approximated as uniform linear motion. This approximation is valid due to the high inertia of vehicles and the short prediction horizon [24]. Let its relative velocity vector be ${\vec{v}}_{rel}$, with magnitude $v_{rel}=||{\vec{v}}_{rel}||$. The angle between this velocity direction and the initial inter-node direction (vector $\vec{SN}$) is denoted as α.
- Channel Randomness: The practical wireless communication boundary is not an ideal disk due to fading, shadowing, and obstacles. To capture these fluctuations, we model the effective communication radius as a random variable: $R_{eff}=R+\xi$, where $\xi ∼N(0,\sigma^{2})$ is a zero-mean Gaussian random variable with standard deviation σ, representing environmental uncertainty. This Gaussian assumption is justified by: (i) the Central Limit Theorem, as multiple independent fading factors combine in V2V channels [25]; and (ii) empirical V2V measurements confirming that shadow fading follows a truncated Gaussian distribution [26].

The above assumptions are reasonable in typical urban road environments: the uniform motion approximation is applicable for short time scales, and Gaussian perturbation can characterize channel uncertainty. This model is specifically valid for the following common scenarios, with corresponding justifications:

Highways and urban arterial roads: Vehicles travel along well-defined lanes with limited lateral movement. The vector $\vec{SN}$ is approximately parallel to the road direction, ensuring that the correlation between the direction angle θ and relative speed $v_{\mathrm{rel}}$ (Section 4.1.2) holds.

Free-flow traffic: Sudden acceleration or deceleration is infrequent; due to vehicle inertia, the constant-velocity approximation over short time windows (≤1 s) is reasonable.

Line-of-sight (LOS) conditions: On highways and arterial roads without large obstacles, LOS propagation dominates, and the Gaussian perturbation model for channel randomness is supported by V2V channel measurements [26].

Short time horizon (≤1 s): This matches the typical beacon interval and the decision timescale of greedy forwarding. Within this window, relative motion can be accurately approximated as uniform linear motion.

Conversely, the model is not applicable to the following common scenarios, with corresponding reasons:

Intersections: Vehicle movement directions change abruptly, and $\vec{SN}$ is no longer parallel to a single road direction. The relationship between θ and $v_{\mathrm{rel}}$ becomes unreliable, and the constant-velocity assumption fails due to turning and stopping.

Frequent lane changes or overtaking: Lateral movements introduce additional relative velocity components that the simplified one-dimensional model cannot capture, and the inter-vehicle distance dynamics become highly nonlinear.

Stop-and-go traffic: Frequent acceleration and braking violate the constant-velocity assumption even over sub-second intervals, and the radial relative speed fluctuates sharply.

Non-line-of-sight (NLOS) conditions: Shadowing from large vehicles or buildings may cause fading distributions that deviate from Gaussian (e.g., heavy tails), and the effective communication radius may experience abrupt changes that a stationary Gaussian perturbation cannot adequately model.

#### 4.1.1. Derivation of Link Survival Probability

Based on the above assumptions, the predicted distance *d*(*t*) between the two nodes at time *t* can be expressed as:

(4) $$d\left(t\right)=d_{0}+v_{rel}\cdot \cos\left(\alpha \right)\cdot t.$$

where $v_{rel}\cdot cos(\alpha )$ is the radial component of the relative velocity along the line connecting the nodes, directly determining whether the distance increases or decreases.

The probability that the link remains alive at time *t*, $P_{link}(t)$ is equivalent to the probability that the predicted distance is less than or equal to the effective communication radius:

(5) $$P_{link}\left(t\right)=P\left(d\left(t\right)\leq R_{eff}\right)=\;P\left(d_{0}+v_{rel}\cdot \cos\left(\alpha \right)\cdot t\leq R+\xi \right).$$

Rearranging the inequality yields:

(6) $$P_{link}\left(t\right)=P\left(\xi \geq d_{0}+v_{rel}\cos\left(\alpha \right)t-R\right).$$

Since ξ follows a standard normal distribution, using its cumulative distribution function Φ(·), the above equation can be expressed as:

(7) $$P_{link}\left(t\right)=1-\Phi \left(\frac{d_{0}+v_{rel}\cos\left(\alpha \right)t-R}{\sigma }\right)=\Phi \left(\frac{R-d_{0}-v_{rel}\cos\left(\alpha \right)t}{\sigma }\right).$$

#### 4.1.2. Correlation Between the Movement Direction Angle *θ* and Model Parameter

To connect the direction angle θ (defined in Section 3.1) with the parameters of the link lifetime model, it is necessary to examine how θ influences the relative speed $v_{\mathrm{rel}}$ and the angle α.

In typical road scenarios(where vehicles travel along lanes and communication mostly occurs between front and rear vehicles in the same lane), the vector $\vec{SN}$ is parallel to the road direction. Under this condition, the correlations between θ and the two parameters are analyzed as follows:

- Relation between θ and $v_{rel}$: The magnitude of the relative velocity is $v_{rel}=∥{\vec{v}}_{n}-{\vec{v}}_{s}∥$. When two vehicles travel in the same direction (small θ), $v_{rel}$ equals the absolute speed difference, which is typically small. When two vehicles travel in opposite directions (large θ), $v_{rel}$ is approximately equal to the sum of the two speeds, which is generally large. Thus, there is a strong correlation between θ and $v_{rel}$: 0 ≤ θ≤π/2 corresponds to a small $v_{rel}$, while π/2<θ<π corresponds to a large $v_{rel}$.
- Relation between θ and α: When vehicles travel in the same direction, ${\vec{v}}_{\mathrm{rel}}$ is parallel to the road. Consequently, α can only take the values 0 (diverging) or π (converging), depending on the relative speed magnitude and front-rear position of *N* with respect to *S*. The same applies to vehicles traveling in opposite directions, though in that case the vehicles typically converge before meeting (α=π) and diverge afterward (α=0). Regardless of whether the vehicles are moving in the same or opposite directions, $|v_{\mathrm{rel}}cos(\alpha )|=v_{\mathrm{rel}}$ holds true.

### 4.2. Correlation Analysis Between Model and Protocol Design

Equation (7) reveals that the decay rate of $P_{\mathrm{link}}(t)$ over time is governed by the term $v_{\mathrm{rel}}cos(\alpha )t$. This key quantity lies at the heart of the three core mechanisms introduced in Section 3.

#### 4.2.1. Theoretical Basis for Direction-Aware Neighbor Classification

The direction-aware classification (Section 3.1) categorizes neighbors into a co-directional set 0<θ≤π/2 and a reverse-direction set π/2<θ<π based on θ, and prioritizes the selection of co-directional nodes. As inferred from the correlation in Section 4.1.2, co-directional nodes correspond to a small $v_{\mathrm{rel}}$, while reverse-direction nodes correspond to a large $v_{\mathrm{rel}}$.

Substituting this relationship into Equation (7) for analysis:

- For co-directional nodes, $v_{\mathrm{rel}}$ is small, so $|v_{\mathrm{rel}}cos(\alpha )|=v_{\mathrm{rel}}$ is also small. Whether the vehicles are diverging (α=0) or converging (α=π), the attenuation or growth of the numerator $R-d_{0}-v_{\mathrm{rel}}cos(\alpha )t$ is slow, and the link survival probability remains at a high level or improves rapidly within the time window.
- For reverse-direction nodes, $v_{\mathrm{rel}}$ is large, resulting in a large $|v_{\mathrm{rel}}cos(\alpha )|$. If the vehicles are diverging (α=0), the term $R-d_{0}-v_{\mathrm{rel}}cos(\alpha )t$ decays rapidly, driving $P_{\mathrm{link}}(t)$ toward zero almost immediately. If they are converging (α=π), the expression $R-d_{0}+v_{\mathrm{rel}}t$ grows quickly, but this apparent improvement is deceptive due to the large relative speed. The two vehicles pass each other in an extremely short time and then move away from each other, leading to an extremely short actual link survival time that cannot meet the requirements of reliable communication.

Hence, by prioritizing co-directional neighbors, the classification scheme effectively selects candidates with small $v_{\mathrm{rel}}$, thereby maximizing the expected link lifetime. Although the co-directional set may occasionally include diverging vehicles, their small relative speed ensures a gradual degradation, which is still far superior to the instantaneous failure of reverse-direction nodes.

#### 4.2.2. Rationale for the Adaptive Greedy Forwarding Region

Traditional GPSR restricts candidate next hops to nodes satisfying d(N,D)<d(S,D), which corresponds to region E in Figure 3. When the network is sparse or the topology changes rapidly, region E may contain no nodes at all. In such cases, the protocol falls back to perimeter forwarding mode—effectively treating the current hop as having no usable link, i.e., $P_{\mathrm{link}}(t)=0$.

VANET-GPSR+ relaxes this constraint by considering all neighbors located in the half-plane in front of S toward the destination D, i.e., region E∪R. From the perspective of the link lifetime model, this expansion addresses the problem of candidate availability: it increases the probability of finding a potential relay when the initial distance $d_{0}$ is large (close to R). However, availability does not guarantee suitability—the enlarged region may contain many reverse-direction nodes whose $P_{\mathrm{link}}(t)$ is extremely low. Thus, the expansion must be coupled with direction-aware classification: it first ensures that at least one candidate exists, and then the classification step filters out those with small $v_{\mathrm{rel}}$. This combined mechanism guarantees that the benefit of a larger search region is not offset by selecting low-quality candidates.

#### 4.2.3. Indirect Contribution of Path Deviation Angle Selection

The path deviation angle $\varphi_{\mathrm{NSD}}$ (defined in Section 3.3) quantifies how much the route from S through N to D deviates from a straight line. While this criterion does not directly optimize the single-hop link survival probability $P_{\mathrm{link}}(t)$, it contributes to end-to-end performance by shaping the geometric conditions of subsequent hops.

Selecting a node with a small $\varphi_{\mathrm{NSD}}$ implies a more direct path toward the destination. In road-constrained environments, this typically leads to shorter initial distances $d_{0}^{\left(i+1\right)}$ in the following hop and increases the likelihood that subsequent vehicles continue traveling along the same road direction, thereby keeping $v_{\mathrm{rel}}$ small in later hops. This notion of “path smoothness” helps suppress the cumulative growth of $v_{\mathrm{rel}}$ across multiple hops, ultimately enhancing end-to-end reliability at the path level.

### 4.3. Summary

The link lifetime probability model directly correlates vehicle movement direction with link stability, providing a unified theoretical explanation for the three core mechanisms of VANET-GPSR+. It reveals that direction-aware neighbor classification enhances link survival probability by minimizing the radial component of relative speed, justifies the adaptive region expansion, and supports the indirect contribution of path deviation angle selection. Unlike existing models that rely on complex mobility prediction, the proposed model uses only directional information from periodic beacons, incurring negligible computational overhead—aligning perfectly with the lightweight design philosophy of VANET-GPSR+.

## 5. Simulation Experiments and Performance Evaluation

To comprehensively evaluate the performance of the VANET-GPSR+ protocol, a series of simulation-based comparative experiments were designed and implemented in this section. The experiments aimed to verify the following core hypotheses: (1) VANET-GPSR+ outperforms mainstream comparative protocols in key performance metrics including PDR and average end-to-end delay; (2) the protocol exhibits favorable robustness under different network conditions (e.g., varying node density and vehicle mobility); (3) compared with existing improved GPSR variants, VANET-GPSR+ maintains lower resource overhead, which is consistent with its lightweight design goal and more suitable for resource-constrained VANET scenarios.

### 5.1. Simulation Environment and Parameter Settings

Experiments were conducted on the NS-3.35 network simulation platform, with vehicle movement trajectories generated by the microscopic traffic simulation software SUMO 1.15.0 (Simulation of Urban Mobility) to realize co-simulation. The simulation adopted real road topologies and vehicle movement patterns conforming to the Intelligent Driver Model (IDM). Other simulation parameters are shown in Table 2.

### 5.2. Baseline Performance Comparison and Analysis

In this section, the protocol performance under different vehicle densities was evaluated with standard parameters (1.0 s beacon period, 300 m communication radius)

#### 5.2.1. Packet Delivery Rate

**Figure 4:**
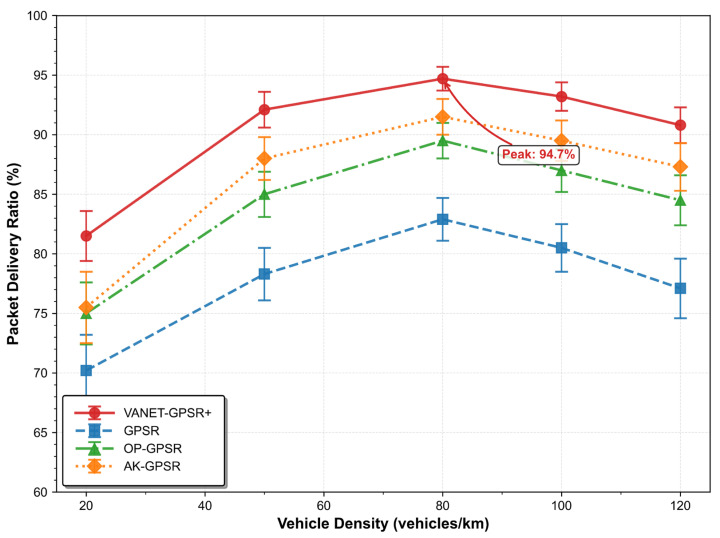
Comparison of Packet Delivery Ratio under Different Vehicle Densities.

Table 3 and Figure 4 illustrate how each protocol’s PDR varies with vehicle density. VANET-GPSR+ achieves the highest performance across all scenarios: 92.1% at 50 vehicles/km (moderate density), 94.7% at 80 vehicles/km (high density), and 81.5% at 20 vehicles/km (sparse density)—the highest across all density ranges. Its core mechanisms (direction-aware neighbor classification and adaptive forwarding region) ensure stable links and path continuity even in sparse networks, enabling more reliable routing than competitors regardless of traffic conditions.

In contrast, AK-GPSR benefits from its clustering mechanism, achieving 91.5% PDR at 80 vehicles/km (significantly higher than the original GPSR) by isolating routing from individual node mobility. However, its performance degrades to 75.5% at 20 vehicles/km (only slightly above OP-GPSR’s 75.0%), as clustering requires sufficient nearby vehicles to form valid groups—unfeasible in sparse networks.

OP-GPSR adopts a distinct optimization approach by integrating node load, link quality, and mobility into local routing decisions. Its PDR ranges from 75.0% to 89.5% across tested densities, consistently outperforming the original GPSR—highlighting the value of comprehensive metrics beyond geographic distance.

The original GPSR exhibits the lowest performance, with PDR fluctuating between 70.2% and 82.9% due to its distance-only routing rule. This confirms that relying solely on geographic distance is insufficient for effective routing in highly dynamic VANETs.

#### 5.2.2. Average End-to-End Delay

**Figure 5:**
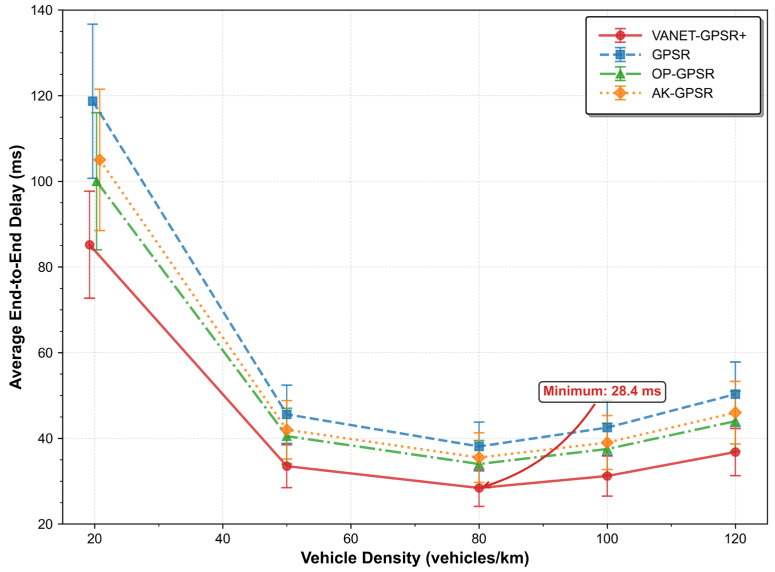
Comparison of Average End-to-End Delay under Different Vehicle Densities.

Table 4 and Figure 5 compare the end-to-end packet delivery latency of each protocol. VANET-GPSR+ again shows the best performance, achieving the lowest end-to-end delay of 28.4 ms at the optimal vehicle density of 80 vehicles/km. This advantage stems from three core mechanisms.

First, direction-aware neighbor classification (Section 3.1) gives priority to vehicles moving in the same direction. As shown by the link lifetime model in Section 4, this reduces the probability of link breakage, which in turn lowers the number of retransmissions and effectively cuts the delivery delay.

Second, the adaptive greedy forwarding region (Section 3.2) expands the search space for candidate next hops in sparse networks, preventing the protocol from falling back prematurely to inefficient perimeter forwarding. This avoids path detours and extra hops caused by routing holes, directly reducing propagation delay.

Third, the path deviation angle criterion (Section 3.3) selects the next hop that minimizes the angular deviation toward the destination. By prioritizing directional consistency, it builds more stable and efficient multi-hop routes. Stable links reduce route reconstructions and retransmissions triggered by topology changes, which also contributes to a higher packet delivery ratio and lower end-to-end delay.

Despite its structural stability, AK-GPSR fails to match this performance, with latency ranging from 35.5 ms to 105.0 ms—slightly higher than OP-GPSR in most scenarios. This is attributed to the inherent limitation of its clustering mechanism: while clustering forms stable node groups, packet forwarding often requires inter-cluster hops, which introduce additional transmission steps and processing latency that offset the stability-induced gains.

OP-GPSR achieves balanced latency performance, with values ranging from 34.0 ms to 100.0 ms. By avoiding congested or low-quality links, it reduces queuing delays and retransmissions. Nevertheless, it lags behind VANET-GPSR+ due to its lack of emphasis on maintaining the overall straightness of the routing path.

The traditional GPSR performs the worst, with latency fluctuating drastically from 38.1 ms to 118.7 ms and exhibiting the widest confidence intervals among all tested protocols. This high variability stems from its distance-only next-hop selection strategy, which disregards node movement direction and link quality, leading to erratic routing paths and frequent retransmissions. In dynamic VANET environments, this approach inevitably results in elevated latency.

#### 5.2.3. Comparative Analysis of Resource Occupancy Among Protocols

**Figure 6:**
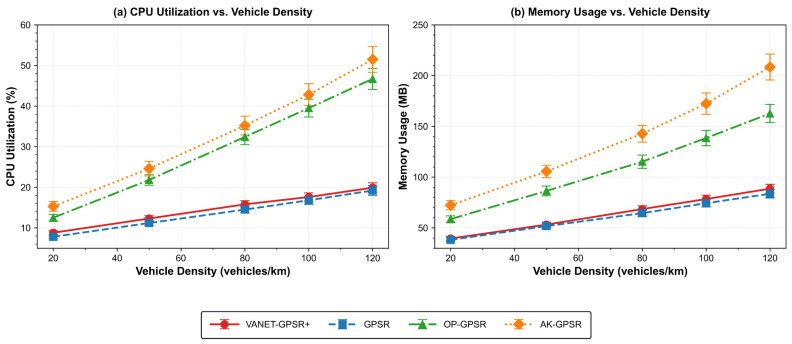
CPU Utilization and Memory Usage of Each Protocol under Different Vehicle Densities.

Table 5 and Table 6, together with Figure 6, compare the CPU utilization and memory usage of each protocol across varying vehicle densities. These results reflect the computational complexity of each protocol, supplement performance metrics, and reveal the trade-off between routing efficiency and resource usage. They also reinforce the lightweight design principle of VANET-GPSR+.

Measurement environment: All CPU and memory measurements were conducted on a Linux server (2 CPU cores, 1 GB RAM) using standard tools (‘top’, ‘/proc/stat’). Values are averages over 10 independent runs per configuration on the same hardware, serving as a relative complexity comparison across protocols.

VANET-GPSR+ achieves high resource efficiency, consistent with its strong forwarding performance. At a medium–high density of 80 vehicles/km, its CPU utilization is 15.8%, just 1.3 percentage points above baseline GPSR (14.5%). This small overhead comes from the direction-aware classification mechanism: upon beacon reception, each node computes motion-vector angles with neighbors and updates neighbor classification tables. Because forwarding decisions are confined to the same-direction neighbor subset, the extra computation is largely offset, leading to a minimal net CPU increase.

In memory footprint, VANET-GPSR+ uses only 6.2% more memory than GPSR—a result of storing motion vectors for direction-based classification. Compared to protocols that maintain multidimensional state information, VANET-GPSR+ consumes substantially less memory, underscoring its fit for resource-limited vehicular environments and validating its lightweight design.

OP-GPSR, in contrast, shows markedly higher resource consumption. Its multi-criteria decision model requires real-time computation and storage of multiple parameters, yielding CPU utilization about 2.05 times that of VANET-GPSR+ and memory usage about 1.68 times higher. Such overhead exceeds the demands of lightweight design and would complicate deployment in realistic VANET scenarios.

Among all tested protocols, AK-GPSR incurs the highest resource overhead, sharply contrasting with VANET-GPSR+. Its clustering mechanism imposes a considerable computational burden—CPU utilization about 2.23 times that of VANET-GPSR+—and maintaining cluster structures demands extensive data storage, resulting in memory usage about 2.08 times higher. These features run counter to the fundamental requirements of lightweight routing in VANETs.

Overall, VANET-GPSR+ consistently balances excellent forwarding performance with low resource consumption across a wide range of vehicle densities. Its distinct lightweight advantage makes it well suited to the resource-constrained, highly dynamic nature of vehicular networks, further confirming the soundness and practicality of the proposed design.

### 5.3. Parameter Sensitivity Analysis and Robustness Verification

#### 5.3.1. Impact of Beacon Period

**Figure 7:**
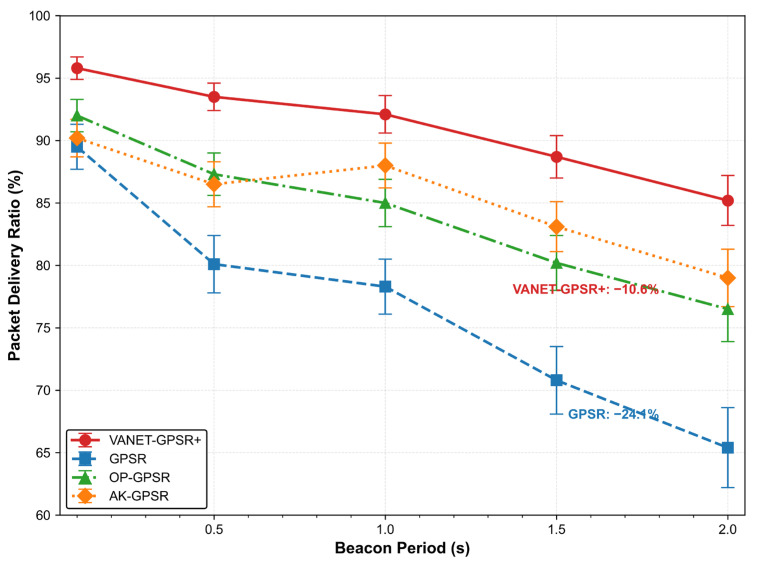
Impact of Beacon Period on PDR.

The interval at which vehicles broadcast beacon messages affects both protocols overhead and the freshness of neighbor information. To assess how each protocol tolerates outdated information, we varied the beacon period from 0.1 s to 2.0 s in the baseline urban scenario (50 vehicles/km, 300 m radio range) and measured the resulting changes in PDR. All protocols were evaluated under identical parameter configurations (same beacon period range, same mobility, same traffic patterns) without any per-protocol optimization. As shown in Table 7 and Figure 7, PDR degrades for all protocols as the beacon interval lengthens, but the rate of degradation differs markedly. These differences reflect each algorithm’s inherent tolerance to staleness in neighbor state information.

VANET-GPSR+ exhibits the strongest robustness. When the beacon period increases from 0.1 s to 2.0 s, its PDR drops from 95.8% to 85.2%—a decline of only 10.6 percentage points, the smallest among all protocols. This result confirms a key premise of the protocol: vehicle movement direction has greater temporal persistence than instantaneous position. Even when position updates are slightly delayed, the directional trend inferred from consecutive beacons remains sufficiently accurate for neighbor classification and forwarding decisions.

GPSR, by contrast, proves most sensitive to information freshness. Its PDR falls by 24.1 percentage points over the same range—a direct consequence of relying solely on instantaneous distance. Once position information becomes stale, the nearest-distance decision rule fails immediately.

OP-GPSR experiences a moderate decline of 15.5 percentage points. Its multi-criteria model depends on several rapidly varying parameters (node load, link quality, etc.). As the beacon interval grows, these inputs become less representative, degrading the overall quality of the next-hop selection.

AK-GPSR exhibits a different behavior. Although its PDR at short intervals (0.1 s) is not the highest, at long intervals (2.0 s) it retains 79.0%—substantially above both GPSR and OP-GPSR. This stems from its clustering mechanism, which builds a relatively stable virtual topology. Once clusters are formed, routing becomes insensitive to small-scale movements of individual nodes, reducing dependence on frequent beacon updates. The same mechanism, however, explains its suboptimal performance at short intervals: forming effective clusters takes time, so the protocol cannot immediately exploit high-frequency updates.

#### 5.3.2. Impact of Communication Radius

**Figure 8:**
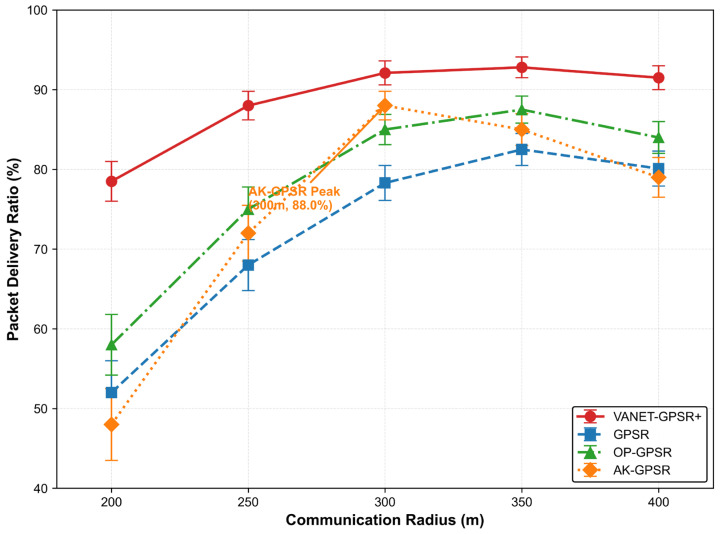
Impact of Communication Radius on PDR.

Communication radius is a critical parameter affecting network connectivity in VANETs. Table 8 and Figure 8 show the PDR achieved by each protocol as the radius varies. VANET-GPSR+ demonstrates strong environmental adaptability. In sparse networks (*R* = 200 m), its adaptive forwarding region strategy maintains a PDR of 78.5%—well above the other protocols. In dense networks (*R* = 400 m), direction-based neighbor screening limits the PDR drop to only 0.6 percentage points from its peak, yielding the flattest performance curve. These results confirm that the protocol’s core mechanisms effectively mitigate both connectivity shortfalls and channel contention.

AK-GPSR exhibits a pronounced inverted-U curve. Its PDR peaks at 88.0% when *R* = 300 m but falls sharply to 48.0% at 200 m and 79.0% at 400 m. This behavior stems from its dependence on forming clusters of optimal density; under extreme conditions, the overhead of cluster management outweighs its benefits, causing substantial performance degradation.

OP-GPSR performs steadily but offers only marginal gains. Its multi-criteria calculation becomes an efficiency bottleneck when the neighbor count grows large. GPSR consistently lags behind, underscoring its heavy reliance on basic network connectivity.

The spatial analysis confirms that VANET-GPSR+ maintains effective performance over a wider range of communication radii, reinforcing the soundness of its adaptive and direction-aware design.

### 5.4. Summary of Findings

The experimental results presented in Section 5 demonstrate that VANET-GPSR+ consistently outperforms the baseline protocols across key performance metrics while maintaining lower resource consumption. Its robustness is evidenced by two observations: the protocol exhibits the slowest performance degradation when beacon updates are delayed, and it sustains efficient routing paths even under limited connectivity. These findings align consistently with the design principles outlined in Section 3 and the theoretical model developed in Section 4, forming a complete chain from conceptual design through mathematical analysis to empirical validation. The collective evidence confirms the effectiveness and advancement of the proposed protocol.

## 6. Conclusions

This paper proposes and validates VANET-GPSR+, a lightweight direction-aware routing enhancement protocol tailored for highly dynamic Vehicular Ad Hoc Networks (VANETs). The protocol integrates three core mechanisms: direction-aware neighbor classification, adaptive greedy forwarding region expansion, and path deviation angle-based next-hop selection, with theoretical underpinnings provided by a probabilistic link lifetime model.

Simulation results demonstrate that VANET-GPSR+ improves the PDR by 16.3% and reduces end-to-end delay by 27.5% compared with the standard GPSR protocol. It also outperforms OP-GPSR and AK-GPSR across diverse network conditions, confirming its superior adaptability to dynamic VANET scenarios. Resource consumption analysis further reveals that its CPU and memory overhead is only marginally higher than that of GPSR, while being approximately 50% and 40% lower than those of OP-GPSR and AK-GPSR, respectively—effectively validating the resource efficiency of its lightweight design.

These results confirm that relying only on directionality—a fundamental feature of VANET nodes—achieves significant performance gains with negligible extra overhead. The protocol retains GPSR’s inherent stateless and distributed characteristics, requiring only basic vector operations on existing periodic beacons. As such, it offers an efficient, reliable, and resource-friendly routing solution for highly dynamic VANET environments.

Future work will focus on five key directions: extending the direction-aware framework to intersection scenarios, validating the protocol in real-world testbed environments, investigating the impact of varying connected vehicle penetration rates on protocol performance, integrating lightweight machine learning techniques (e.g., adaptive threshold tuning, context-aware switching) into VANET-GPSR+ to improve adaptability under dynamic conditions, while preserving real-time and OBU resource constraints, and extending the comparative evaluation to include other MCDM-based direction-aware protocols. 

## Figures and Tables

**Table 1:** Comparison of representative GPSR-based routing protocols.

Protocol	Category	Core Method	Key Limitations
GPSR	Baseline	Greedy (closest to destination) + perimeter forwarding	Lacks direction awareness; link instability; routing voids
OP-GPSR	MCDM	Multi-criteria cost function (distance, mobility, load, link quality)	High computational burden; multi-parameter storage
MM-GPSR	MCDM	Cumulative communication duration + minimal-angle perimeter	Parameter tuning (λ); periodic duration calculations
DVA-GPSR	MCDM	Weighted function (heading angle, speed variation, node density)	Periodic weight recalculation
W-PAGPSR	MCDM	Weighted greedy (distance, reliable node density, link duration, direction)	Continuous parameter collection; weight update overhead
WA-GPSR	MCDM	Reliable communication area + multi-criteria selection	Multi-parameter fusion; complex computation
W-GPSR	MCDM	Greedy link weight factor (GLWF) + mobility prediction	Mobility prediction overhead
PA-GPSR	Table-based	Deny table (DT) + recently sent table (RST)	Additional memory overhead
AK-GPSR	Clustering	Adaptive K-medoids unsupervised learning	High signaling for cluster maintenance; sensitive to topology changes
GOA-WOA	Clustering	Bio-inspired hybrid optimization (grasshopper + whale)	Complex optimization; high computational cost

**Table 2:** Core Simulation Parameter Configuration.

Parameter Category	Detailed Configuration and Value
Simulation Platform	NS-3.35, integrated with SUMO 1.15.0
Scenario Map	Highway: 5 km two-way six-lane Urban grid: 9 × 9 blocks with cross intersections, 200 m spacing between adjacent blocks
Communication Model	PHY/MAC: IEEE 802.11p (ITS-G5) Frequency: 5.89 GHz Bandwidth: 10 MHz Transmission power: 20 dBm Reception sensitivity: −89 dBm Communication radius (variable): default 300 m
Propagation model	Two-Ray Ground Reflection
Vehicle Mobility	Speed: 80–120 km/h (highway), 30–60 km/h (urban road) Density: 20, 50, 80, 100, 120 vehicles per kilometer Mobility model: Generated by SUMO, following lane-keeping and IDM car-following models
Data Traffic	10 randomly selected pairs of CBR (Constant Bit Rate) flows Packet size: 512 Bytes Sending rate: 4 packets/s
Beacon Mechanism (Routing update interval)	Period (variable): default 1.0 s Content: Node ID, current position coordinates, current velocity vector
Comparative Protocols	GPSR, AK-GPSR, OP-GPSR
Performance Metrics	Packet Delivery Rate End-to-End Delay CPU Utilization Memory Usage Impact of Beacon Period on PDR Impact of Communication Radius on PDR
Statistical Method	For each configuration (e.g., a specific density, beacon interval, or radius), 10 independent runs are performed on the highway scenario and 10 independent runs on the urban grid scenario (20 runs in total). Each run uses a different random seed for both mobility (SUMO) and network traffic (NS-3). Simulation time per run is 300 s. Results are averaged over the 20 runs, and 95% confidence intervals are computed based on these independent samples.

**Table 3:** Comparison of PDR of Each Protocol under Different Vehicle Densities(%).

Vehicle Density (Vehicles/km)	VANET-GPSR+	GPSR	OP-GPSR	AK-GPSR
20	81.5 ± 2.1	70.2 ± 3.0	75.0 ± 2.6	75.5 ± 3.0
50	92.1 ± 1.5	78.3 ± 2.2	85.0 ± 1.9	88.0 ± 1.8
80	94.7 ± 1.0	82.9 ± 1.8	89.5 ± 1.5	91.5 ± 1.5
100	93.2 ± 1.2	80.5 ± 2.0	87.0 ± 1.8	89.5 ± 1.7
120	90.8 ± 1.5	77.1 ± 2.5	84.5 ± 2.1	87.3 ± 2.0

**Table 4:** Average End-to-End Delay of Each Protocol under Different Vehicle Densities (Unit: ms).

Vehicle Density (Vehicles/km)	VANET-GPSR+	GPSR	OP-GPSR	AK-GPSR
20	85.2 ± 12.5	118.7 ± 18.0	100.0 ± 16.0	105.0 ± 16.5
50	33.5 ± 5.0	45.6 ± 6.8	40.5 ± 6.5	42.0 ± 6.8
80	28.4 ± 4.3	38.1 ± 5.7	34.0 ± 5.5	35.5 ± 5.8
100	31.2 ± 4.7	42.5 ± 6.4	37.5 ± 6.0	39.0 ± 6.3
120	36.8 ± 5.5	50.3 ± 7.5	44.0 ± 7.0	46.0 ± 7.3

**Table 5:** CPU Utilization Comparison under Different Vehicle Densities (%).

Vehicle Density (Vehicles/km)	VANET-GPSR+	GPSR	OP-GPSR	AK-GPSR
20	8.8 ± 0.5	7.8 ± 0.4	12.5 ± 0.8	15.3 ± 1.2
50	12.3 ± 0.7	11.2 ± 0.6	21.8 ± 1.3	24.6 ± 1.8
80	15.8 ± 0.9	14.5 ± 0.8	32.4 ± 1.9	35.2 ± 2.3
100	17.6 ± 1.0	16.8 ± 1.0	39.5 ± 2.2	42.8 ± 2.7
120	19.9 ± 1.2	19.2 ± 1.2	46.7 ± 2.6	51.5 ± 3.2

**Table 6:** Memory Usage Comparison under Different Vehicle Densities (MB).

Vehicle Density (Vehicles/km)	VANET-GPSR+	GPSR	OP-GPSR	AK-GPSR
20	39.5 ± 2.0	38.2 ± 1.9	58.6 ± 3.2	72.3 ± 4.5
50	53.2 ± 2.5	51.7 ± 2.5	86.4 ± 4.8	105.6 ± 6.2
80	68.5 ± 3.2	64.5 ± 3.1	115.2 ± 6.5	142.8 ± 8.3
100	78.4 ± 3.8	74.3 ± 3.7	138.5 ± 7.6	172.4 ± 10.5
120	88.6 ± 4.3	83.6 ± 4.2	162.7 ± 8.9	208.5 ± 12.8

**Table 7:** Comparison of PDR under Different Beacon Periods(%).

Beacon Period (s)	VANET-GPSR+	GPSR	OP-GPSR	AK-GPSR
0.1	95.8 ± 0.9	89.5 ± 1.8	92.0 ± 1.3	90.2 ± 1.5
0.5	93.5 ± 1.1	80.1 ± 2.3	87.3 ± 1.7	86.5 ± 1.8
1.0	92.1 ± 1.5	78.3 ± 2.2	85.0 ± 1.9	88.0 ± 1.8
1.5	88.7 ± 1.7	70.8 ± 2.7	80.2 ± 2.2	83.1 ± 2.0
2.0	85.2 ± 2.0	65.4 ± 3.2	76.5 ± 2.6	79.0 ± 2.3

**Table 8:** Comparison of PDR under Different Communication Radius (%).

Communication Radius (m)	VANET-GPSR+	GPSR	OP-GPSR	AK-GPSR
200	78.5 ± 2.5	52.0 ± 4.0	58.0 ± 3.8	48.0 ± 4.5
250	88.0 ± 1.8	68.0 ± 3.2	75.0 ± 2.8	72.0 ± 3.5
300	92.1 ± 1.5	78.3 ± 2.2	85.0 ± 1.9	88.0 ± 1.8
350	92.8 ± 1.3	82.5 ± 2.0	87.5 ± 1.7	85.0 ± 2.0
400	91.5 ± 1.5	80.1 ± 2.2	84.0 ± 2.0	79.0 ± 2.5

## Data Availability

The raw data supporting the conclusions of this article will be made available by the authors on request.
